# Associations between Physical Fitness, Bone Mass, and Structure in Older People

**DOI:** 10.1155/2020/6930682

**Published:** 2020-07-15

**Authors:** A. Moradell, A. Gómez-Cabello, A. Gómez-Bruton, B. Muniz-Pardos, J. Marín Puyalto, A. Matute-Llorente, A. Gónzalez-Agüero, I. Ara, J. A. Casajús, G. Vicente-Rodríguez

**Affiliations:** ^1^GENUD (Growth, Exercise, NUtrition and Development) Research Group, Universidad de Zaragoza, Zaragoza, Spain; ^2^Instituto Agroalimentario de Aragón -IA2- (CITA-Universidad de Zaragoza), Spain; ^3^Red española de Investigación en Ejercicio Físico y Salud en Poblaciones Especiales (EXERNET), Spain; ^4^Department of Physiatry and Nursing, Faculty of Health and Sport Science (FCSD), University of Zaragoza, Ronda Misericordia 5, 22001 Huesca, Spain; ^5^Centro de Investigación Biomédica en Red de Fisiopatología de la Obesidad y Nutrición (CIBERObn), Spain; ^6^Centro Universitario de la Defensa, Zaragoza, Spain; ^7^Centro de Investigación Biomédica en Red de Fragilidad y Envejecimiento Saludable (CIBERFes), Spain; ^8^GENUD Toledo Research Group, Universidad de Castilla la Mancha, Toledo, Spain; ^9^Faculty of Health, Department of Physiatry and Nursing, University of Zaragoza, Zaragoza, Spain

## Abstract

The main aim was to analyse the associations between several physical fitness variables and bone parameters in a sample of elderly people. 129 participants (94 females and 35 males, 76.2 ± 5.4 y) from the EXERNET cohort of Zaragoza (Spain) were included in the study. Physical fitness was assessed using the Senior Fitness Test Battery. Peripheral quantitative computed tomography (pQCT) at the tibia and dual-energy X-ray absorptiometry (DXA) at the hip and lumbar spine were used to assess bone and muscle parameters. Partial correlations were used to describe the associations between fitness and bone parameters. A stepwise regression analysis was used to determine the influence of fitness variables on bone parameters. In males, significant correlations were found between lower body strength and agility with bone total mineral density (Tt.BMD) (*r* = 0.41 and -0.50) and cortical thickness (*r* = 0.40 and -0.50, respectively) and walking speed with total and cortical density (*r* = −0.41 and -0.40, respectively), all measured at tibia (all *p* < 0.05). Regarding DXA, neck areal bone mineral density (aBMD) correlated with flexibility (*r* = −0.37) and walking speed (*r* = 0.39) and Ward's triangle with walking speed (*r* = 0.39). Agility predicted Tt.BMD and cortical thickness (*r*^2^ change = 24.8% and 23.0%), while walking speed predicted cortical bone mineral density (*r*^2^ change = 19.5%) (all *p* < 0.05). Females showed correlations between balance and total hip aBMD (*r* = 0.27) and trochanter aBMD (*r* = 0.25). Balance predicted trochanter (*r*^2^ change = 4.2%) and total hip aBMD (*r*^2^ change = 4.9%) (both *p* < 0.05). In conclusion, bone mass in elderly males seems to be more influenced by physical fitness than in females, being agility and walking speed the variables showing greater associations. Other variables should be taken into account in females for future research.

## 1. Background

Worldwide life expectancy has more than doubled since 1900, and most people can expect to live into their 60s and beyond [1]. In Europe, a twofold increase in the proportion of people aged 65 and older is expected to occur between 2010 and 2050 and the number of people aged 85 years and older is projected to rise from 14 million in 2010 to 40 million by 2050 [1].

Aging results in a progressive and generalized impairment of several bodily functions, an increased vulnerability to environmental challenges, and a growing risk of disease and death [2]. The aging process entails a decrease of both muscle and bone tissue, which may increase the incidence of osteoporosis and the risk of suffering falls and fractures [3]. However, as Harridge and Lazarus reflected in a recent review [4], it is time to look beyond this aging model. They showed that active seniors showed superior health and well-being, and an optimized aging process [4] when compared with their inactive pairs. These improvements seemed to be associated with their physical fitness levels.

Previous research has studied physical fitness, defined as a set of attributes that are either health- or skill-related [5], linked to bone mass in different populations such as children [6], adolescents [7], adults [8], or people with disabilities [9]. However, most of the studies have analysed bone parameters through dual-energy X-ray absorptiometry (DXA), which evaluates only areal bone mineral density (aBMD). The use of other devices assessing volumetric BMD or bone structural parameters such as peripheral quantitative computed tomography (pQCT), high-resolution pQCT, or magnetic resonance imaging (MRI) is not common in previous studies; therefore, the use of these devices may contribute to a deeper knowledge of the relationship between fitness level and bone health.

In relation to elderly people, some of the most widely studied physical fitness-related variables associated with bone health are aerobic fitness, maximal muscle strength, and balance [10–13]. It is worth noting that other fitness-related variables such as agility and flexibility have been studied to a lesser extent. The large methodological differences between studies make conclusions difficult to establish; thus, the fitness-related variables associated with a greater bone health remain under debate.

Therefore, the aim of this study was to analyse the association between different physical fitness tests and bone variables as measured by pQCT and DXA, in a sample of noninstitutionalized Spanish elderly individuals.

## 2. Materials and Methods

### 2.1. Study Design and Participants

The study was performed in the framework of the elderly EXERNET study (Exernet Elder 3.0), a multicentric study performed between 2008 and 2017 on a representative sample of Spanish seniors from different regions of the country. The inclusion criteria for the EXERNET study were as follows: noninstitutionalized participants over 65 years and not suffering from dementia or cancer, as described elsewhere [14]. For this study, only data from those seniors participating in the study of the city of Zaragoza (Spain) having complete information of the incorporated measurements of both DXA and pQCT were used in the analysis. Consequently, the final sample for the present study consisted of 129 participants (94 females and 35 males).

Personal information was collected through a structured and validated questionnaire (which included two specific questions about daily sitting time and daily sedentary hours) [15], followed by anthropometric, bone, and fitness assessments. Written informed consent was obtained from all the included participants. The study protocol was performed in accordance with the Helsinki Declaration of 1961 (revised in Fortaleza, 2013) and was approved by the Clinical Research Ethics Committee of Aragón (18/2008) and by the Hospital Universitario Fundación de Alcorcón (16/50).

### 2.2. Anthropometric Measurements

A portable stadiometer with 2.10 m maximum capacity and 1 mm error margin (Seca 711, Hamburg, Germany) was used to measure height. A body composition analyser with a 200 kg maximum capacity and a ±50 g error margin (TANITA BC-418MA, Tanita Corp., Tokyo, Japan) was used to measure the body mass. Individuals removed shoes and heavy clothes before weighing. Body mass index (BMI) was calculated by dividing weight (kg) by squared height (m^2^).

### 2.3. Bone Mass Measurements

#### 2.3.1. Bone Assessment by Peripheral Quantitative Computed Tomography

Peripheral quantitative computed tomography measurements were taken at four sites (4%, 14%, 38%, and 66%) of the tibia length using a Stratec XCT-2000 L pQCT scanner (Stratec Medizintechnik, Pforzheim, Germany). To ensure machine stability, the pQCT device undertook a daily quality control using a phantom (Stratec Medizintechnik, Pforzheim, Germany).

The nondominant tibia was selected for the measurements. Participants were seated in a stationary chair, adjusted to an appropriate height to ensure the leg was appropriately placed in a straight position. The tibia length from the distal end of the medial malleolus to the medial knee joint cleft was measured. A coronal computed radiograph (scout view) was performed to manually locate a reference line on the distal end of the tibia. The measurement sites were located proximal to this reference line by a distance corresponding to 4% (distal tibia) and 38% (diaphyseal tibia), as previously described [16]. For muscle area, the measurement site was at 66% of the tibia length, where the largest calf diameter is typically located. In this study, we considered the following bone parameters: total bone mineral content (Tt.BMC), total bone mineral area (Tt.Ar), total bone mineral density (Tt.BMD) (all of them at 4% and 38% of the tibia), trabecular bone mineral density (Tb.BMD), cortical bone mineral density (Ct.BMD), cortical bone thickness (Ct.Th), and muscle cross sectional area (MCSA) at 66% of the tibia. Bone strength was established with respect to resistance to torsion (polar stress strain index in mm^3^ (SSIp)) and bending, as fracture load X (N), with respect to the *x*-axis, as it has been described in detail elsewhere [17]. Both of them were measured at the 38% of the tibia.

#### 2.3.2. Bone Assessment by Dual-Energy X-Ray Absorptiometry

A DXA scanner (QDR 4500-Explorer, Hologic Corp., Software version 12.4, Bedford, Massachusetts, USA) was used to evaluate aBMD at the lumbar spine (mean *L*_1_-*L*_4_) and proximal region of the femur (total hip, femoral neck, Ward's triangle, and trochanter). Additionally, whole body and regional lean mass (kg) were also analysed. All DXA scans were completed using the same device and software and performed by the same technician who had been fully trained in the operation of the scanner, the positioning of subjects, and the analysis of results, according to the manufacturer's instructions.

### 2.4. Physical Fitness Assessments

Prior to testing, training workshops were organized to harmonize the assessment of physical fitness among researchers. For this report, six tests modified from the “Senior Fitness Test Battery” (tests: 2, 3, 4, 6) [18] and the “Eurofit testing battery for older”(tests: 1, 5) [19] were selected, excluding those involving upper-body limbs. The tests were always performed in the same order to ensure that all participants performed the fitness battery under the same conditions.

1. Balance test (Flamingo's test). The maximum standing time (maximum 60 s) on one foot with both hands on the hip was assessed. The test was performed twice with the right and left feet alternatively. The best result obtained among the four attempts was recorded
2. Lower body strength (LBS) test (chair stand test). The number of full stands from a seated position that could be completed in 30 s with arms folded across the chest was determined. This test was performed once
3. Flexibility of the lower extremities (chair sit-and-reach test). The number of centimetres, between the extended and gathered fingers and the tip of the toe (plus or minus, considering if the participants did or did not surpass the tip of the toe, respectively). The test was performed once with each leg, selecting the best attempt for further analyses
4. Agility/dynamic balance (8-foot up-and-go test). Each participant was required to get up from a seated position, walk 2.45 m, and return to a seated position as fast as possible. The test was performed twice and the best result was recorded
5. Maximum walking speed (brisk walking test). This test consisted of a 30 m walking sprint performed as fast as possible. The test was performed twice with at least one minute of rest between repetitions. The best result was recorded
6. Aerobic capacity (6-minute walk test). The distance that participants could walk in 6 minutes around a circuit of 46 m was recorded. Only one attempt was permitted

### 2.5. Statistical Analysis

The Statistical Package for the Social Sciences (SPSS) v. 20.0 for Windows (SPSS, Inc., Chicago, Illinois, USA) was used to analyse the data. All the analyses were performed with the sample divided by sex. Normality of the sampling distribution was assumed as explained by the central limit theorem [20]. To compare descriptive variables between genders, *t*-tests were performed. Mean and SD were reported for all anthropometric, bone, and fitness variables. Partial correlation analysis adjusting by age, height, and subtotal lean (for DXA variables) and age, tibia length, and muscle mass (for pQCT variables) was used to determine associations between fitness and bone variables. Only those fitness and bone variables showing statistical correlations were included in a stepwise regression analysis in order to determine the predictive values of the fitness variables on bone mass. Variables used to adjust in the correlation analysis were included by the enter method in the regression. All the analyses were repeated adjusting by sitting time and walking hours. Standardized *β*, change in *r*^2^, and overall *r*^2^ of the model were reported. Statistical significance was set at level *p* < 0.05 in all tests.

## 3. Results

The final sample included 129 participants (35 males and 94 females) aged 65 and older (76.2 ± 5.4 y). The anthropometric characteristics, physical fitness, and bone parameters of the whole sample and stratified by sex are displayed in Table 1.

### 3.1. Associations between Physical Fitness Variables and Bone Structural and Strength Parameters (pQCT)

#### 3.1.1. Males

No associations were found between bone parameters at 4% of the tibia length and physical fitness variables, so they were not presented in the tables.

Regarding 38% of the tibia length, results are presented in Table 2. Tt.BMD was correlated to LBS (*r* = 0.411), agility (*r* = −0.503), and walking speed (*r* = −0.414). Ct.BMD was correlated to walking speed (*r* = −0.398) and Ct.Th to LBS (*r* = 0.395) and agility (*r* = −0.503).

#### 3.1.2. Females

No associations were found between the physical fitness variables and bone tibia variables measured by pQCT for females (all *p* > 0.05).

### 3.2. Associations between Physical Fitness Variables and Bone Mass Parameters (DXA)

#### 3.2.1. Males

Neck aBMD was correlated with lower body flexibility (*r* = −0.396) and walking speed (*r* = −0.393). Ward's aBMD was positively correlated with walking speed (*r* = 0.390) (all *p* < 0.05; Table 2). Trochanter aBMD, lumbar spine aBMD, and total hip aBMD were not correlated with any fitness variable (*p* > 0.05).

#### 3.2.2. Females

Balance showed a positive correlation with trochanter aBMD and total hip aBMD (*r* = 0.253 and *r* = 0.267, respectively; both *p* < 0.05; Table 3). Neck aBMD, lumbar spine aBMD, and Ward's aBMD did not show correlations with fitness variables (*p* > 0.05).

Bone variables not showing associations were not shown in tables.

No different results were found when analyses were adjusted by sitting and walking hours for pQCT nor for DXA in neither of the sexes.

### 3.3. Influence of Physical Fitness Variables on Bone Parameters

Predictive values of fitness in bone variables are presented in Table 4.

#### 3.3.1. Males

Regarding 38% of the tibia length, total bone mineral density and cortical thickness were partially explained by agility (change in *r*^2^ = 0.248 and 0.230, respectively; both *p* < 0.05, Table 4). Moreover, walking speed predicted cortical bone mineral density (change in *r*^2^ = 0.195; *p* < 0.05, Table 4). LBS and lower flexibility were not significant, so they were not included in any model.

#### 3.3.2. Females

Balance explained trochanter aBMD (change in *r*^2^ = 0.042, *p* < 0.05) and total hip aBMD (change in *r*^2^ = 0.049, *p* < 0.05), as it has been shown in Table 4.

No significant different predictive values were found when analyses were adjusted by sitting time and walking hours.

## 4. Discussion

The main findings of the present study were as follows: agility and walking speed showed the greatest influence with bone mass and structure in males, while balance was associated with areal bone mineral density in females.

Due to the possible segmentation of the bone that pQCT provides, different researchers have studied the relationship between bone tissues and several factors like muscle mass [21], tibia length, or physical activity, among others [22]. In the present study, bone parameters measured by pQCT have shown to be partially explained by physical fitness in elderly people. Specifically, agility and walking speed were positively associated with bone mineral density and thickness at 38% of the tibia length suggesting that good levels of physical fitness might help to preserve Ct.BMD during aging. These results could be partially explained because cortical bone is more associated with the stiffness of the bone and more influenced by mechanical forces than trabecular bone, which is more metabolically active and could be more influenced by other factors such as hormones [23]. Previous literature shows conflictive results. In our line, Barbour et al. observed a positive association between the time to perform five chair stands and cortical volumetric BMD at 33% of the tibia length [24]. However, further studies found that gait speed was not related to any variable of the tibia as measured by pQCT [21, 24].

Bone variables measured by DXA showed a significant association between femoral neck and Ward's triangle aBMD with agility; however, no significant results were found when applying the linear regressions, which suggest that in males, physical fitness does not influence aBMD variables after accounting for age, height, and lean mass. Results suggest that bone in male elders may have changes in structure not affecting the aBMD [25], which probably occurs due to the physical activities in which they were involved [26]. This fact is of great relevance, due to DXA measurements may mask positive changes on bone parameters in this specific gender, and therefore, it may lead to inappropriate decisions in the exercise programs prescribed for the elderly men.

In females, our data did not present remarkable associations between physical fitness and bone variables measured by pQCT. It is worth considering that most of the published studies examining fitness and bone in elderly population have mainly used DXA, with few studies using pQCT. This limitation makes conclusions derived from previous literature difficult to draw. A previous study using pQCT in females showed that power from the lower limbs predicted a 6.6% of the strength strain index at the tibial mid-shaft [27], not in line with our results but, suggesting that other strength variables such as power should be interesting for the study.

When evaluating aBMD, analyses did reveal a small predictive contribution of balance to trochanter and total hip aBMD after adjusting by age, height, and lean mass. In this line, controversial results have been found in previous literature. While some studies found a positive correlation between balance and femoral neck [28], lumbar and femoral regions [12], and trochanter aBMD [29], other researchers did not find any of these associations neither in females [30] nor in males [29]. A possible explanation for these results could be that, in comparison with males, older females of our study were more involved in organized activities such as yoga, pilates, tai chi, or maintenance gymnastics, where balance-based exercises and unilateral balance training have a greater importance. Thus, a potentially increased neuromuscular capability during this type of exercises may explain bone characteristics on the hip region as it was explained by other authors [31].

Flexibility and aerobic capacity did not show associations with bone parameters in our model. A possible reason for this might be that specific physical exercises to improve these variables do not entail high muscle contractions or impacts which could lead to the activation of the osteogenic process. To the best of our knowledge, this study is one of the pioneering studies examining the association between flexibility and bone mass. In relation to the previous research focusing on the relationship between aerobic capacity and bone mass in elderly individuals, unclear conclusions arise from contrasting results. A study with a Portuguese sample of 401 males and 401 females found associations between endurance measured by the 6-minute walk test with hip aBMD in both genders [12], while other studies did not find any associations between aerobic capacity, measured with a treadmill and aBMD at any site [11, 32]; results that are in line with those found in our study.

The results found in this study show that bone mass is less influenced by physical fitness in females than in males, probably because there are different bone remodelling mechanisms between sexes.

The importance of physical fitness in bone mass found in males when bone was measured by pQCT may be masked in elderly females due to the complexity of female-related issues [33]. Some aspects as age of menarche, number of births, or age of menopause may be more important than physical fitness in terms of bone health in this specific population. Our findings suggest the importance of maintaining high levels of agility and walking speed in elderly males, as they may contribute to guarantee an increased bone health at this late stage of life. Probably, other factors should be taken into consideration in future research such as muscle power, physical activity, sedentary behaviours, food supplements, and vitamin intake to improve bone mass in females. Moreover, further research should implement exercise programs to study if fitness-related enhancement might really evoke improvements on bone parameters at these ages.

Some strengths and limitations of this study should be highlighted. The present study has a cross-sectional design, reflecting associations but not revealing causality. Further research including larger sample sizes is required to verify these results in representative populations. Although we controlled for several potential confounders, we cannot be certain that other confounders such as dietary calcium intake, smoking, or genetic variations influenced our observations. However, some strengths like harmonized assessments, well-instructed researchers, and validated physical fitness tests should also be considered. Finally, the inclusion of both pQCT and DXA devices to evaluate bone parameters is another strength of this study.

## 5. Conclusion

In conclusion, pQCT bone parameters are more influenced by physical fitness in males than females, showing agility and walking the greatest associations. Although DXA is the gold standard diagnosis method for bone health, pQCT should be taken in consideration for a deeper insight of bone and fitness associations in this population.

## Figures and Tables

**Table 1:** Descriptive variables of the sample.

	Whole sample (*n* = 129)	Males (*n* = 35)	Females (*n* = 94)
Anthropometrics			
	Mean ± SD	Mean ± SD	Mean ± SD
Age (years)	76.2 ± 5.4	76.2 ± 6.1	76.2 ± 5.1
Height (cm)	156.6 ± 8.6	166.8 ± 5.9	152.4 ± 5.3^∗^
Weight (kg)	68.4 ± 11.7	77.4 ± 10.2	64.7 ± 10.2^∗^
BMI (kg/cm^2^)	28.9 ± 5.1	27.9 ± 3.5	29.3 ± 5.6
Fitness variables			
Balance (s)	23.9 ± 5.1	25.3 ± 23.6	23.2 ± 21.4
Lower body strength (reps.)	14.2 ± 3.3	13.7 ± 3.8	14.4 ± 3.0
Lower body flexibility (cm)	−8.2 ± 11.1	−12.0 ± 11.5	−6.6 ± 10.5^∗^
Agility (s)	6.0 ± 1.4	5.8 ± 1.2	6.1 ± 1.4
Gait speed (s)	17.3 ± 3.6	15.8 ± 3.3	17.9 ± 3.6^∗^
Aerobic capacity (m)	504.1 ± 108.7	542.7 ± 97.9	488.8 ± 109.4^∗^
pQCT variables			
Tt.BMC 4% (g)	2.79 ± 0.77	3.84 ± 0.55	2.40 ± 0.37^∗^
Tt.Ar 4% (mm^2^)	1118.71 ± 169.64	1325.24 ± 150.71	1069.26 ± 115.46^∗^
Tt.BMD 4% (mg/cm^3^)	244.06 ± 47.27	291.05 ± 36.51	226.56 ± 38.04^∗^
Tb.BMD 4% (mg/cm^3^)	194.44 ± 39.56	223.39 ± 32.29	183.66 ± 36.61^∗^
Tt.BMC 38% (g)	3.07 ± 0.73	3.82 ± 0.94	2.79 ± 0.36^∗^
Tt.Ar 38% (mm^2^)	377.68 ± 71.04	437.56 ± 100.44	355.15 ± 36.91^∗^
Tt.BMD 38% (mg/cm^3^)	808.57 ± 98.80	868.84 ± 78.07	786.13 ± 96.59^∗^
Ct.BMD 38% (mg/cm^3^)	1134.70 ± 41.39	1153.70 ± 33.84	1127.63 ± 41.87^∗^
Ct.Th 38% (mm)	4.38 ± 0.91	5.18 ± 1.04	4.09 ± 0.64^∗^
Fracture load X 38% (N)	3323.97 ± 956.87	4627.43 ± 746.93	2852.50 ± 454.49^∗^
SSIp 38% (mm^3^)	1471.25 ± 417.23	2032 ± 324.34	1262.36 ± 196.44^∗^
MCSA 66%(mm^2^)	5943.72 ± 1136.91	7169.67 ± 969.92	5489.15 ± 814.00^∗^
DXA variables			
Trochanter aBMD (g/cm^2^)	0.627 ± 0.122	0.729 ± 0.126	0.583 ± 0.090^∗^
Neck aBMD (g/cm^2^)	0.666 ± 0.112	0.744 ± 0.101	0.632 ± 0.090^∗^
Ward's triangle aBMD (g/cm^2^)	0.472 ± 0.126	0.516 ± 0.120	0.453 ± 0.124^∗^
Total hip aBMD (g/cm^2^)	0.801 ± 0.140	0.918 ± 0.139	0.752 ± 0.108^∗^
Lumbar spine aBMD (g/cm^2^)	0.925 ± 0.191	1.070 ± 0.200	0.863 ± 0.149^∗^
Subtotal lean mass (kg)	38.752 ± 8.212	49.022 ± 5.204	34.317 ± 4.408^∗^

SD: standard deviation; reps: repetitions; pQCT: peripheral quantitative computed tomography; DXA: dual-energy X-ray absorptiometry; aBMD: areal bone mineral density; Tt.BMC: total bone mineral content; Tt.Ar: total bone area; Tt.BMD: total bone mineral density; Tb.BMD: trabecular bone mineral density; Ct.BMD: cortical bone mineral density; Ct.Th: cortical thicknes; MCSA: muscle area; SSIp: polar stress strain index. ^∗^Statistical significant differences between sexes (*p* < 0.05).

**Table 2:** Partial correlation coefficients between bone mass variables and physical fitness in males, for age, tibia length, and muscle area as possible confounders.

	Balance	LB strength	LB flexibility	Agility	Walking speed	Aerobic capacity
pQCT variables						
Tt.BMD 38% (mg/cm^3^)	0.047	**0.411**	0.202	**-0.503**	**-0.414**	0.184
Ct.BMD 38% (mg/cm^3^)	-0.141	0.295	0.198	-0.346	**-0.398**	0.003
Ct Th 38% (mm)	0.081	**0.395**	0.218	**-0.503**	-0.175	0.261
DXA variables						
Neck aBMD (g/cm^2^)	-0.184	-0.313	**-0.396**	0.271	**0.393**	-0.199
Ward's triangle aBMD (g/cm^2^)	-0.054	-0.189	-0.312	0.261	**0.390**	-0.096

LB: lower body; pQCT: peripheral quantitative computed tomography; Tt.BMD: total bone mineral density; Ct.BMD: cortical bone mineral density; Ct.Th: cortical thickness; aBMD: areal bone mineral density. Significant correlations are in bold numbers.

**Table 3:** Partial correlation coefficients between bone mass variables and physical fitness in females, for age, tibia length, and muscle area as possible confounders.

	Balance	LB strength	LB flexibility	Agility	Walking speed	Aerobic capacity
DXA variables							
Trochanter aBMD (g/cm^2^)	**0.253**	0.019	0.152	-0.150	0.167	0.130
Total hip aBMD (g/cm^2^)	**0.267**	-0.053	0.195	-0.053	-0.111	0.074

LB: lower body; pQCT: peripheral quantitative computed tomography; DXA: dual-energy X-ray absorptiometry; aBMD: areal bone mineral density. Significant correlations are in bold numbers.

**Table 4:** Bone mass physical fitness significant predictive values from the stepwise linear regression model for each variable in males and females.

		Balance	LB strength	Agility	Walking speed
Males					
Tt.BMD 38% (mg/cm^3^)	Overall (*R*^2^)	—	—	0.364	—
	Change *r*^2^	—	—	0.248	—
	Standardized	—	—	-0.621	—
	Unstandardized	—	—	-47.736	—
Ct.BMD 38% (mg/cm^3^)	Overall (*R*^2^)	—	—	—	0.37
	Change *r*^2^	—	—	—	0.195
	Standardized	—	—	—	-0.557
	Unstandardized	—	—	—	-6.937
Ct.Th 38% (mm)	Overall (*R*^2^)	—	—	0.418	—
	Change *r*^2^	—	—	0.230	—
	Standardized	—	—	-0.598	—
	Unstandardized	—	—	-0.575	—
Females					
Trochanter aBMD (g/cm^2^)	Overall (*R*^2^)	0.261	—	—	—
	Change *r*^2^	0.042	—	—	—
	Standardized	0.247	—	—	—
	Unstandardized	0.001	—	—	—
Total hip aBMD (g/cm^2^)	Overall (*R*^2^)	0.281	—	—	—
	Change *r*^2^	0.049	—	—	—
	Standardized	0.265	—	—	—
	Unstandardized	0.001	—	—	—

LB: lower body; Tt.BMD: total bone mineral density; Ct.BMD: cortical bone mineral density; Ct.Th: cortical thickness; aBMD: areal bone mineral density. Stepwise regression model controlling for age, object length, and muscle area (for pQCT variables) and age, height, and subtotal lean (for DXA variables) as possible confounders.

## Data Availability

No data were used to support this study.
